# An Experimental Multi-Target Tracking of AM Radio-Based Passive Bistatic Radar System via Multi-Static Doppler Shifts

**DOI:** 10.3390/s21186196

**Published:** 2021-09-15

**Authors:** Xueqin Zhou, Hong Ma, Hang Xu

**Affiliations:** 1 School of Electronic Information and Communications, Huazhong University of Science and Technology, Wuhan 430074, China; xuhang90@foxmail.com; 2 Department of Electrical and Electronic Engineering, College of Engineering Technology, Hubei University of Technology, Wuhan 430068, China

**Keywords:** PBR, CBMeMBer, doppler, tracking

## Abstract

This paper presents a description of recent research and the multi-target tracking in experimental passive bistatic radar (PBR) system taking advantage of numerous non-cooperative AM radio signals via multi-static doppler shifts. However, it raises challenges for use by multiple spatially distributed AM radio illuminators for multi-target tracking in PBR system due to complex data association hypotheses and no directly used tracking algorithm in the practical scenario. To solve these problems, after a series of key array signal processing techniques in the self-developed system, by constructing a nonlinear measurement model, the novel method is proposed to accommodate nonlinear model by using the unscented transformation (UT) in Gaussian mixture (GM) implementation of iterated-corrector cardinality-balanced multi-target multi-Bernoulli (CBMeMBer). Simulation and experimental results analysis verify the feasibility of this approach used in a practical PBR system for moving multi-target tracking.

## 1. Introduction

Passive bistatic radar (PBR) is a subset of bistate radars receiving non-cooperative transmitters of opportunity scattered by potential targets. Research on PBR has attracted extensive attention because of well-known advantages, such as no additional frequency channel allocation, lower costs, and lower probability of being detected with respect to active radars. Although, PBR systems have a long history, there are not enough operational systems.

Of all the transmitters of opportunity available in the PBR systems, very high frequency/ultra high frequency (VHF/UHF) bands represent some of the most attractive for surveillance purposes, such as analog television (ATV) [1,2], digital television-terrestrial (DTV) [3,4], frequency modulation (FM) radio [5], digital audio/video broadcasting (DAB/DVB) [6,7,8,9,10]. However, relatively little interest has been shown in the high frequency (HF) band (3–30 MHz) due to the propagation complexity and low range resolution. Especially, the external illuminators in the HF band have excellent range coverage, propagation over the horizon, and stealth target detection. Some preliminary and pioneering HF-PBR works have been carried out. In the PBR system [11,12], Thomas et al. from University College London performed an analysis using the HF digital radio Mondiale (DRM)signal as transmitter of opportunity. The two-dimensional target localization, using a linear frequency modulated continuous waveform from a non-cooperative OTH radar located in Longreach, Australia, with a bandwidth of 10 kHz, is presented in [13].

In fact, compared with other opportunity illuminators in the HF band, commercial amplitude modulation (AM) broadcast signal sources have the advantages of high transmitter power, larger numbers, and wider coverage. Due to the propagation complexity and bandwidth limited, little attention is paid to AM radio signal for PBR system. The research development on the practical PBR system named “Sugar Tree” was the first using the AM broadcast signal as a passive radar illuminator to detect the missiles launched near the broadcast emitter site. Although this system was shut down decades ago, the performance and method details have not yet been elaborated [14]. One target was tracked by using HF-AM radio signals in passive system [15]. We note that to date, there has been little convincing experimental evidence that AM radio based PBR systems, in particular, can be used for the purpose of multi-target tracking.

Actually, using the AM broadcast signal as an illuminator for air surveillance raises new challenges for the multi-target tracking algorithm in PBR system. First, range resolution is not available or often of extremely poor quality. For example, the regular effective AM radio broadcast bandwidth is 5 kHz so that the practical radar range resolution is up to 60km. That would be unacceptable for the PBR systems. Second, the signal-to-noise ratio (SNR) of echo signal is particularly low and there exists the clutter interference, strong direct path interference, and the missing alarm. Third, in order to track the target, the system must observe the target’s Doppler history for an extended time before there is sufficient information. Furthermore, an additional type of data association uncertainty arises due to the use of multiple AM radio broadcast signals, i.e., not only is it unknown which measurement belongs to which target, there is also the problem that the data association hypotheses arise from the number of AM radio broadcast illuminators. In this paper, we report the newly experimental development of a PBR system using multiple AM radio broadcast signals as a transmission source.

Since the PBR system can typically collect bistatic range, time-of-arrival (TOA), direction-of-arrival (DOA), and Doppler shift from the received signals, an alternative choice is to use multi-static Doppler shift as measurements for multi-target tracking. Target estimation using only Doppler shift measurements is an old problem studied in different contexts [16,17,18]. To achieve the target location accuracy, multiple transmitters can be utilized simultaneously in the system. Therefore, target tracking in the AM radio based PBR system is a multi-sensor fusion problem. Some works on localization and tracking multi-targets in a multi-sensor radar system via multi-static Doppler-shift measurements only have been studied recently. It is shown in [19] that a multi-sensor Bernoulli filter with multi-static Doppler-only measurements contaminated by unknown probability of false alarms and missed detections is able to perform multiple target tracking via numerical simulation examples. An algorithm for fusing data from a constellation of RF sensors detecting cellular emanations with the output of a multi-spectral video tracker to localize and track a target with a specific cell phone is present in [20]; the results show that it is possible to track multiple targets using Doppler differential measurements. However, few field experiments have been performed to demonstrate the target tracking performance in similar scenarios.

In this paper, we went a step further and cast close-in moving multi-target tracking via multi-static Doppler shifts in the practical PBR system by multiple spatially distributed AM radio illuminators. We made use of a cardinality-balanced multi-target multi-Bernoulli (CBMeMBer)-based random finite set (RFS) approach to sequentially estimate targets, with iterated-corrector (IC) iterating the filter update stage. Compared to data association methods, a CBMeMBer-based Bayesian algorithm can avoid complex association processes; it also has significant advantages in less cardinality bias and smaller complexity in solving multi-target tracking problems. There are two implementations of the CBMeMBer filter, Gaussian mixtures (GM) and sequential Monte Carlo (SMC). Contrary to SMC implementation, GM implementation provides more reliable state estimates and a lower computation complexity in an efficient way [21]. However, the GM-CBMeMBer filter does not directly accommodate a nonlinear measurement model. To solve this, we extended GM implementation of the CBMeMBer filter to update the procedure by using unscented transformation (UT). Simulation performance demonstrates the analysis, and the real data from experimental results indicate feasibility performance of the proposed IC-UT-GM-CBMeMBer filter. The system described in this paper was constructed on one of the simplest and lowest cost architectures; therefore, the main contribution of this paper is that the proposed multi-target tracking method can provide a reference for similar PBR systems.

The rest of this paper is organized as follows. The description of the AM Radio based PBR system and multi-target tracking formulation are described in Section 2. Section 3 provides the proposed IC-UT-GM-CBMeMBer filter. Simulation and field experimental implementation are given in Section 4. Finally, conclusion and possible future directions are drawn in Section 5.

## 2. Problem Formulation

### 2.1. System Description

**Figure 1:**
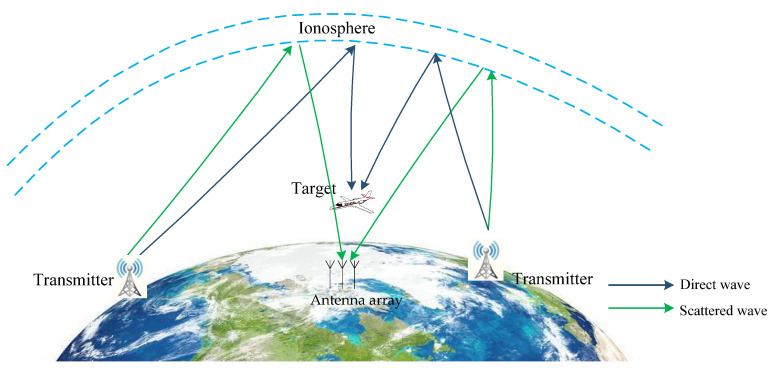
Plane geometry of AM-radio based-sky-surface wave PBR system.

Supposing the ionosphere is homogeneous and spherically symmetric, the bistatic plane geometry of an AM-radio-based sky-surface wave PBR system in the scenario can be simplified as shown in Figure 1 (two transmitters are shown explicitly simplistically). In the system, we exploited one receiving antenna array approximately 20 m above ground level located over-the-horizon (farther than 1000 km from the noncooperative transmitters generally) at Hubei province of China, which is equipped with a uniform circular array (UCA) with 16 antennas.

The direct wave (emitter-to-receiver) and the illumination wave (emitter-to-target) are reflected from the ionosphere, while the echo wave (target-to-receiver) was via line-of-sight (LOS) propagation in the surveillance area.

**Figure 2:**
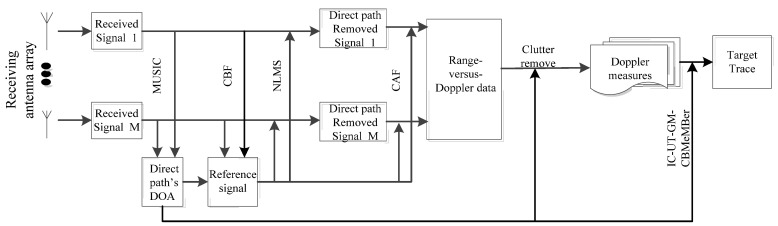
Signal processing diagram.

To obtain multi-static Doppler measurements in the PBR system, some key techniques in array signal processing are summarized in a block diagram sketched in Figure 2. Similar to traditional passive radar, surveillance and reference channels are needed to receive target echoes and reference signal, respectively. The DOA of direct wave can be obtained by the multiple signal classification (MUSIC) algorithm from the reference channel, which is then used to clean the reference signal by using conventional beamforming (CBF) technology. After scanning the surveillance channel by normalized least mean square (NLMS) technology to obtain the echo signal, we calculated the cross-ambiguity function (CAF) of the direct path signal and the scattered signal to estimate range vs. Doppler shift of the targets. Finally, the time delay and Doppler shift of the targets after clutter removal were estimated.

Furthermore, the greatest limitation on tracking target performance in the self-developed experimental PBR system is the interference and clutter in the received signal, for example, dense direct path interference and the DOA of echo signal with very low SNR under masking effects. Although the classic DOA estimation and clutter suppression methods have been studied over the past decade [22,23], most of them are unsuitable for processing HF-AM radio signal. We adopted the method of reference [24] by building a single-snapshot virtual array signal. After extending the single-snapshot virtual array signal to multi-snapshots and the MUSIC algorithm, the clutter interference could be suppressed significantly, and the desired echo signal was enhanced simultaneously. More details of signal processing and improvement can be found in [24]. Finally, the excellent Doppler shift information of targets’ corresponding time can be provided on a 2-D time vs. Doppler map.

### 2.2. CBMeMBer Filter

The CBMeMBer filter is first introduced to solve the aforementioned tracking problem in the PBR system in this section [21].

At time *k*, there are *N*(*k*) target states $X_{k}=\left\{x_{k,1},\cdots ,x_{k,N\left(k\right)}\right\}\subseteq ℱ\left(\chi \right),$ which denote space of finite subsets of χ. Given a target $x_{k}$ at time *k*, it is either detected in the surveillance area with probability $p_{D,k}\left(x_{k}\right)$ and generates a Bernoulli RFS $\Theta_{k}\left(x_{k}\right)$ with likelihood function $g_{k}(\cdot |x_{k})$, or it is missed with probability $1-p_{D,k}(x_{k})$. Given a multi-target state $X_{k}$, each $x_{k}\in X_{k}$ either continues to exist at time *k* + 1 with probability $p_{S}(x_{k+1})$ and moves to a new state $x_{k+1}$ with target transition equation $f_{k+1|k}(x_{k})$ or dies with probability $1-p_{S,k}(x_{k+1})$. Thus, given a target with state $x_{k}\in X_{k}$ at time *k*, its behavior time *k* + 1 is modeled by the Bernoulli RFS $S_{k+1|k}(x_{k})$, and $\Gamma_{k+1}$ denotes the multi-Bernoulli RFS of new births at time *k* + 1. The multi-target state is modeled as [25].

(1) $$X_{k+1}=[\cup \;S_{k+1|k}(x_{k})]\cup \Gamma_{k+1}$$

Similarly, there are $N_{k}^{}$ measurements $Z_{k}^{}=\{z_{k,1}^{}{}_{,}\ldots ,z_{k,N_{k}^{}}^{}\}$, each taking values in an observation space at time *k*. In addition, the received measurement also contains a set of missing alarms or clutter that can be modeled as a Poisson RFS ${Κ}_{k}$. Thus, multi-target observation at time *k* + 1 is modeled as finite sets [26].

(2) $$Z_{k+1}^{}=[\underset{x\in X_{k+1}}{\cup }\Theta_{k+1}^{}(x)]\cup {Κ}_{k+1}^{}$$

where $\Theta (x_{k+1})$ is a Bernoulli RFS that is generated by target state $x_{k+1}\in X_{k+1}$.

A multi-Bernoulli RFS $X^{\left(i\right)}$ on χ is a union of a fixed number M of independent Bernoulli RFSs with existence probability $r^{\left(i\right)}$ and probability density $p^{\left(i\right)}$, $X=\cup_{i=1}^{M}X^{\left(i\right)}$. Moreover, the probability density π is [27]:

(3) $$\pi (\emptyset )=\prod_{j=1}^{M}\left(1-r^{\left(j\right)}\right)$$

(4) $$\pi (\{x_{1},\cdots x_{n}\})=\pi (\emptyset )\sum_{1\leq i_{1}\neq \cdots \neq i_{n}\leq M}\prod_{j=1}^{M}\frac{r^{\left(i_{j}\right)}p^{\left(i_{j}\right)}(x_{j})}{\left(1-r^{\left(i_{j}\right)}\right)}$$

The multi-target bayes recursion propagates in time [27]:

(5) $$\pi_{k+1|k}(X_{k}|Z_{1:k})=\int f_{k+1|k}(X_{k+1}|X)\pi_{k}(X|Z_{1:k})\delta X$$

(6) $$\pi_{k+1}(X_{k+1}|Z_{1:k+1})=\frac{g_{k+1}(Z_{k+1}|X_{k+1})\pi_{k+1|k}(X_{k+1}|Z_{1:k})}{\int g_{k+1}(Z_{k+1}|X)\pi_{k+1|k}(X|Z_{1:k})\delta X}$$

where $f_{k+1|k}(\cdot |\cdot )$ is the multi-target transition density and $g_{k+1}(\cdot |\cdot )$ is the multi-target likelihood.

### 2.3. Multi-Target Tracking Model

Tracking model is one of the major problems needing to be considered in the multi-target system. In this paper, we consider the target tracking scenario performed in a 2D Cartesian coordinate, with the origin point located at a single receiver antenna array; the *x*-axis points East and the *y*-axis points North. Assume that at time *k*, the *i*-th target state is represented by the state vector $x_{k}^{\left(i\right)}=[p_{x,k}^{\left(i\right)}\;v_{x,k}^{\left(i\right)}\;p_{y,k}^{\left(i\right)}\;{v_{y,k}^{\left(i\right)}]}^{T}$, i=1,2,…,N(k) Where N(k) is the number of targets, superscript T denotes the matrix transpose, $p_{k}^{\left(i\right)}={[p_{x,k}^{\left(i\right)}\;p_{y,k}^{\left(i\right)}]}^{T}$ and $v_{k}^{\left(i\right)}=[v_{x,k}^{\left(i\right)}\;{v_{y,k}^{\left(i\right)}]}^{T}$ are the position and velocity of the target, respectively. Each target dynamic motion is followed by a nearly constant velocity model:

(7) $$x_{k+1}^{\left(i\right)}=F_{k}x_{k}^{\left(i\right)}+u_{k}$$

where $u_{k}∼N(u;0,Q_{k})$ is zero-mean white Gaussian process noise with covariance $Q_{k}$. We adopt:

$$F_{k}=\left[\begin{array}{l} A_{0}\;0_{2}\\ 0_{2}\;A_{0} \end{array}\right]Q_{k}=\sigma_{v}^{2}\left[\begin{array}{l} \Delta^{2}I_{2}\;0_{2}\\ 0_{2}\;\Delta^{2}I_{2} \end{array}\right]$$

In which Δ is the sampling interval. $A_{0}=\left[\begin{array}{ll} 1 & \Delta \\ 0 & 1 \end{array}\right]$, $I_{n}$, and $0_{n}$ denote n×n identity and zeros matrices, respectively.

**Figure 3:**
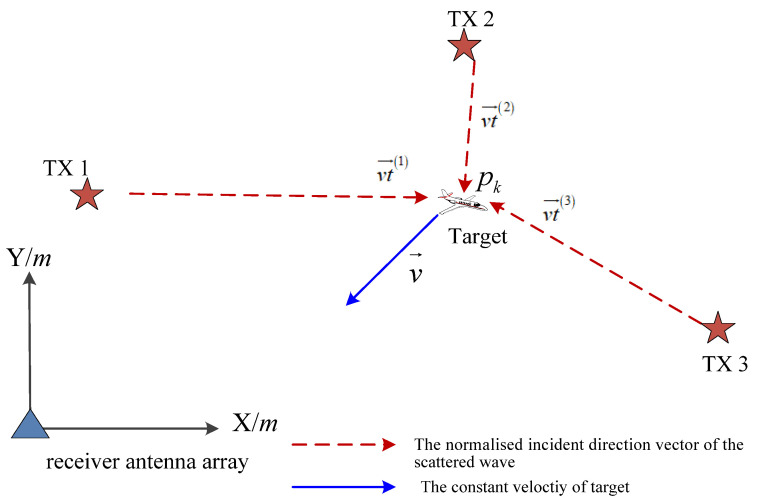
Multiple non-cooperative illuminators of AM radio stations (red pentacle) reflected from ionosphere received by single receiver antenna array (blue triangle) in x-y coordinate.

In the two-dimensional surveillance area, three spatially distributed non-cooperative AM radio illuminators constantly transmit signals with a known carrier frequency $f_{c}^{{}^{\left(i\right)}}$ of the *i*-th AM radio station, *i* = 1 … 3, and the receiver places are at the original point, as illustrated in Figure 3. The direct wave and scattered waves from multiple AM broadcast stations reflected from the ionosphere (three scattered echo waves and one target are shown simplistically) reach the target and receiver simultaneously on the condition that the AM broadcast stations located at $R_{t}^{\left(i\right)}={\left[x_{t}^{\left(i\right)}y_{t}^{\left(i\right)}\right]}^{T}$ are far away (>1000 km) from the receiver antenna array. Doppler shift measurements by *i*-th illumination can be generally modeled as [28]:

(8) $$z_{k}{}^{\left(i\right)}=h^{\left(i\right)}\left(x_{k}\right)+\varepsilon_{k}{}^{\left(i\right)}$$

where:

(9) $$h^{\left(i\right)}\left(x_{k}\right)=-v_{k}^{T}\left[\frac{p_{k}}{\left\|p_{k}\right\|}+\frac{p_{k}-R_{t}^{\left(i\right)}}{\left\|p_{k}-R_{t}^{\left(i\right)}\right\|}\right]\frac{f_{c}^{\left(i\right)}}{c}$$

$\vec{v_{k}}$ is the constant velocity vector of target at time *k*, $\left\|p_{k}\right\|=\sqrt{p_{x,k}{}^{2}+p_{y,k}{}^{2}}$, *c* is the speed of light, $\varepsilon_{k}{}^{\left(i\right)}$ is the measurement noise,$\varepsilon_{k}{}^{\left(i\right)}∼N\left(w;0,\sigma_{w}{}^{2}\right)$.

As the AM radio stations are typically long distance from the receiver antenna array, we approximately considered the direction of the direct wave from the *i*-th illuminator of AM radio station to the receiver antenna array as equal to the direction of the scattered wave from the *i*-th illumination to the target. According to Equation (9), the model can be approximately written as:

(10) $$h^{\left(i\right)}\left(x_{k}\right)=\frac{f_{c}^{\left(i\right)}}{c}\left[-\vec{v_{k}}{\vec{p}}_{k}{}^{\left(i\right)}+\vec{v_{k}}\cdot {\vec{vt}}_{k}{}^{\left(i\right)}\right]$$

Here, ${\vec{p}}_{k}{}^{\left(i\right)}$ is the normalized target position relative to the receiver. ${\vec{vt}}_{k}{}^{\left(i\right)}$ is the normalized incident direction vector of the direct wave from the *i*-th illuminator of the AM radio station to the receiver antenna array, which is independent on the target state and could be easily achieved from DOA estimation of the direct wave, as mentioned before.

Each Doppler-shift subset includes at most one measurement per illuminator and corresponds to the measurements made by multi-targets across all illuminators practically contaminated by false alarms and misdetections. The subsets of a partition are disjointed and comprise measurement space, which is denoted as $Z_{k}^{\left(i\right)}=\left\{\begin{array}{cccc} z_{k,1}^{\left(i\right)} & z_{k,2}^{\left(i\right)} & \cdots & z_{k,N_{k}^{\left(i\right)}}^{\left(i\right)} \end{array}\right\}$, in which *i* is the *i*-th illuminator; $N_{k}^{\left(i\right)}$ is the number of the detection values, including false alarms and misdetection; $z_{k,j}^{\left(i\right)}$ is the *j*-th detection value. Therefore, $Z_{k}^{\left(I\right)}$ is the measurement set involving all *Ns* illuminators at time *k*, and $Z_{1:k}^{\left(I\right)}$ is the time sequence of measurement sets encapsulated $I=\{1,2,\ldots ,N_{S}\}$ illuminator characteristics.

## 3. The Proposed Multi-Target Tracking Method

The GM-CBMeMBer filter has a close-form solution under assumptions of linear Gaussian models that is difficult to implement on the nonlinear measurement models. To overcome this limitation, we extended the GM-CBMeMBer filter to a practical nonlinear measurement model by using unscented transform (UT) techniques [29]. Another straightforward extension of the single sensor CBMeMBer filters to the case of multiple illuminators can be achieved by iterating the filter update stage for each illuminator measurement set. An IC-UT-GM-CBMeMBer filter can be implemented to accommodate a multi-transmitter nonlinear Doppler model. However, this IC-CBMeMBer yields final solutions that depend on the order of the measurement set of illuminators; therefore, the development of efficient algorithms for the scenario case are left for future investigation. Hence, in this section we propose the IC-UT-GM-CBMeMBer filter for multi-target tracking in the PBR system.

### 3.1. IC-UK-GM-CBMeMBer Filter

We supposed that each target follows a linear Gaussian dynamical and observation mode [21], i.e.,

(11) $$f_{k+1|k}(x|\zeta )=N(x;F_{k}\zeta ,Q_{k})$$

(12) $$g_{k+1}(z|x)=N(z;H_{k+1}x,R_{k+1})$$

where $f_{k+1|k}(\cdot |x_{k})$ is a transition function commonly known as Markov shift [30].N( ⋅ ;m,P) denotes a Gaussian density with mean m and covariance P, $F_{k}$ is the state transition matrix, $Q_{k}$ is the process noise covariance, $g_{k+1}(z|x)$ is likelihood function, $H_{k+1}$ is the observation matrix, and $R_{k+1}$ is the observation noise covariance.

A multi-Bernoulli RFS is characterized by a posterior distribution with parameters existence probability $r_{}^{\left(i\right)}$ and probability density $p_{}^{\left(i\right)}$ of the *i*-th hypothesized track, $i=1,\ldots ,M_{k},$ i.e.,$\pi_{k}={\left\{r_{k}^{\left(i\right)},p_{k}^{\left(i\right)}(x_{k})\right\}}_{i=1}^{M_{k}}$, which is comprised of Gaussian mixtures of the form $p_{k}^{\left(i\right)}(x_{k})=\sum_{j=1}^{J_{k-1}^{\left(i\right)}}w_{k}^{\left(i,j\right)}N(x_{k};m_{k}^{\left(i,j\right)},P_{k}^{\left(i,j\right)})$, where $w_{k}^{\left(i,j\right)},m_{k}^{\left(i,j\right)},P_{k}^{\left(i,j\right)}$ denote the weights, means, and covariances of the *j*-th Gaussian component by the sample time *k*.

The Bernoulli filter propagates the posterior $\pi_{k}=\{r_{k}^{},p_{k}^{}(x_{k})\}$ during the whole time in “prediction” and “update” steps. This effectively means that $r_{k}^{}$ and $p_{k}^{}$ must be propagated.

Prediction: At time *k* + 1, spontaneous births are accounted for by appending a birth multi-Bernoulli RFS with components ${\left\{r_{\Gamma ,k+1}^{\left(i\right)},p_{\Gamma ,k+1}^{\left(i\right)}\right\}}_{i=1}^{M_{\Gamma ,k+1}}$ to surviving targets. The total number of predicted hypothesized tracks is $M_{k+1|k}=M_{k}+M_{\Gamma ,k+1}$. The predicted multi-target density is [21]:

$$\pi_{k+1|k}=\{(r_{P,k+1|k}^{\left(i\right)},p_{P,k+1|k}^{\left(i\right)}){\}}_{i=1}^{M_{k}}\cup {\left\{(r_{\Gamma ,k+1}^{\left(i\right)},p_{\Gamma ,k+1}^{\left(i\right)})\right\}}_{i=1}^{M_{\Gamma ,k+1}}$$

where:

(13) $$p_{\Gamma ,k+1}^{\left(i\right)}(x)=\sum_{j=1}^{J_{\Gamma ,k+1}^{\left(i\right)}}w_{\Gamma ,k+1}^{\left(i,j\right)}N(x;m_{\Gamma ,k+1}^{\left(i,j\right)},P_{\Gamma ,k+1}^{\left(i,j\right)})$$

(14) $$r_{P,k+1|k}^{\left(i\right)}=r_{k}^{\left(i\right)}p_{S,k+1}$$

(15) $$p_{P,k+1|k}^{\left(i\right)}(x)=\sum_{j=1}^{J_{k}^{\left(i\right)}}w_{k}^{\left(i,j\right)}N(x;m_{P,k+1|k}^{\left(i,j\right)},P_{P,k+1|k}^{\left(i,j\right)})$$

$$m_{P,k+1|k}^{\left(i,j\right)}=F_{k}m_{k}^{\left(i,j\right)}$$

$$P_{P,k+1|k}^{\left(i,j\right)}=Q_{k}+F_{k}P_{k}^{\left(i,j\right)}F_{k}^{T}$$

Update: In the following, based on nonlinear observations, we propose the unscented transform implementation of the IC-UK-GM-CBMeMBer filter in the update step.

At time *k* + 1, the updated multi-Bernoulli density $\pi_{k+1}$ is formed by multi-Bernoulli RFS of the legacy tracks $(r_{L,k+1}^{\left(i\right)},p_{L,k+1}^{\left(i\right)}){\}}_{i=1}^{M_{k+1|k}}$ and measurement-corrected tracks ${\left\{(r_{U,k+1}(W),p_{U,k+1}(\cdot ;z))\right\}}_{z\in Z_{k}}$ [21], as follows:

$$\pi_{k+1}={\left\{(r_{L,k+1}^{\left(i\right)},p_{L,k+1}^{\left(i\right)})\right\}}_{i=1}^{M_{k+1|k}}\cup {\left\{(r_{U,k+1}(z),p_{U,k+1}(\cdot ;z))\right\}}_{z\in Z_{k+1}}$$

where:

(16) $$r_{L,k+1}^{\left(i\right)}=r_{k+1|k}^{\left(i\right)}\frac{1-p_{D,k+1}}{1-r_{k+1|k}^{\left(i\right)}p_{D,k+1}}$$

(17) $$p_{L,k+1}^{\left(i\right)}=p_{k+1|k}^{\left(i\right)}(x)$$

(18) $$r_{U,k+1}(z)=\frac{\sum_{i=1}^{M_{k+1|k}}\frac{r_{k+1|k}^{\left(i\right)}(1-r_{k+1|k}^{\left(i\right)})\rho_{U,k+1}^{\left(i\right)}(z)}{{\left(1-r_{k+1|k}^{\left(i\right)}p_{D,k+1}\right)}^{2}}}{\kappa_{k+1}(z)+\sum_{i=1}^{M_{k+1|k}}\frac{r_{k+1|k}^{\left(i\right)}\rho_{U,k+1}^{\left(i\right)}(z)}{1-r_{k+1|k}^{\left(i\right)}p_{D,k+1}}}$$

(19) $$p_{U,k+1}(x;z)=\frac{\sum_{i=1}^{M_{k+1|k}}\sum_{j=1}^{J_{k+1|k}^{\left(i\right)}}w_{U,k+1}^{\left(i,j\right)}(z)N(x;m_{U,k+1}^{\left(i,j\right)},P_{U,k+1}^{\left(i,j\right)})}{\sum_{i=1}^{M_{k+1|k}}\sum_{j=1}^{J_{k+1|k}^{\left(i\right)}}w_{U,k+1}^{\left(i,j\right)}(z)}$$

Using unscented transform extends the mean matrix and covariance matrix, respectively,

(20) $$\mu_{k+1}=[m_{k+1|k}0^{\prime }0^{\prime }{]}^{\prime }$$

(21) $$C_{k+1}=\mathrm{diag}(P_{k+1|k},Q_{k+1},R_{k+1})$$

We constructed a set of $2n_{U}+1$ sigma points ${\left\{\chi_{k}^{\left(\ell \right)}\right\}}_{\ell =0}^{L}$ and weights ${\left\{u^{\left(\ell \right)}\right\}}_{\ell =0}^{L}$,$L=2n_{U}$, i.e.,

(22) $$\left\{ \begin{array}{lll} \chi_{k+1}^{\left(\ell \right)}=\mu_{k+1} & u^{\left(\ell \right)}=\frac{\kappa_{U}}{n_{U}+\kappa_{U}} & \ell =0\\ \chi_{k+1}^{\left(\ell \right)}=\mu_{k+1}+{\left(\sqrt{(n_{U}+\kappa_{U})C_{k+1}}\right)}_{\ell } & u^{\left(\ell \right)}=\frac{1}{2(n_{U}+\kappa_{U})} & \ell =1,\cdots ,n_{U}\\ \chi_{k+1}^{\left(\ell \right)}=\mu_{k+1}-{\left(\sqrt{(n_{U}+\kappa_{U})C_{k+1}}\right)}_{\ell } & u^{\left(\ell \right)}=\frac{1}{2(n_{U}+\kappa_{U})} & \ell =n_{U}+1,\cdots ,L \end{array}\right.$$

where $n_{U}$ is the dimension of $\mu_{k}$, and $\kappa_{U}$ is the scaling parameters,$n_{U}+\kappa_{U}\neq 0$.

(23) $$q_{k+1}^{\left(i,j\right)}(z)=N(z_{k+1};z_{k+1|k},S_{K+1})$$

(24) $$\rho_{U,k+1}^{\left(i\right)}(z)=p_{D,k}\sum_{j=1}^{J_{k+1|k}^{\left(i\right)}}w_{k+1|k}^{\left(i,j\right)}q_{k+1}^{\left(i,j\right)}(z)$$

(25) $$m_{U,k+1}^{\left(i,j\right)}(z)=m_{k+1|k}^{\left(i,j\right)}+K_{U,k+1}^{\left(i,j\right)}(z_{k+1}-z_{k+1|k})$$

(26) $$P_{U,k+1}^{\left(i,j\right)}=P_{U,k+1|k}^{\left(i,j\right)}-G_{k+1}S_{k+1}^{-1}{G^{\prime }}_{k+1}$$

(27) $$z_{k+1|k}=\sum_{\ell =0}^{L}u^{\left(\ell \right)}z_{k+1|k}^{\left(\ell \right)}$$

(28) $$K_{U,k+1}^{}=G_{k+1}S_{k+1}^{-1}$$

(29) $$S_{k+1}=\sum_{\ell =0}^{L}u^{\left(\ell \right)}(z_{k+1|k}^{\left(\ell \right)}-z_{k+1|k})(z_{k+1|k}^{\left(\ell \right)}-z_{k+1|k}{)}^{\prime }+R_{k}$$

(30) $$G_{k+1}=\sum_{\ell =0}^{L}u^{\left(\ell \right)}(\chi_{k+1|k}^{\left(\ell \right)}-m_{k+1|k})(z_{k+1|k}^{\left(\ell \right)}-z_{k+1|k}{)}^{\prime }$$

(31) $$z_{k+1|k}^{\left(\ell \right)}=h_{k}(\chi_{k+1}^{\left(\ell \right)}),\;\ell =0,\ldots ,L$$

(32) $$w_{U,k+1}^{\left(i,j\right)}(z)=\frac{r_{k+1|k}^{\left(i\right)}}{1-r_{k+1|k}^{\left(i\right)}}p_{D,k+1}w_{k+1|k}^{\left(i,j\right)}q_{k+1}^{\left(i,j\right)}(z)$$

With the *i*-th illuminator measurement data $Z_{k+1}^{\left(i\right)}$, the filter is obtained by the aforementioned sequential processing of the measurement set of each illuminator with the CBMeMBer filter corrector. The update operator $\Psi_{k+1}{}^{\left(i\right)}$ from $\pi_{k+1|k}^{\left(i\right)}$ to $\pi_{k+1}^{\left(i\right)}=\{r_{k+1}^{\left(i\right)},p_{k+1}^{\left(i\right)}\}$ is [21,31]:

(33) $$\left[\Psi_{k+1}{}^{\left(i\right)}r\right]=\frac{\sum_{i=1}^{M_{k+1|k}}\frac{r_{k+1|k}^{\left(i\right)}\langle p_{k+1|k}{}^{\left(i\right)},g_{k+1}\left(Z^{i}|x\right)p_{D,k+1}\rangle }{1-r_{k+1|k}^{\left(i\right)}\langle p_{k+1|k}{}^{\left(i\right)},p_{D,k+1}\rangle }}{\kappa_{k+1}(Z^{\left(i\right)})+\sum_{i=1}^{M_{k+1|k}}\frac{r_{k+1|k}^{\left(i\right)}\langle p_{k+1|k}{}^{\left(i\right)},g_{k+1}\left(Z^{\left(i\right)}|x\right)p_{D,k+1}\rangle }{1-r_{k+1|k}^{\left(i\right)}\langle p_{k+1|k}{}^{\left(i\right)},p_{D,k+1}\rangle }}$$

(34) $$\left[\Psi_{k+1}{}^{\left(i\right)}p\right]=\frac{\sum_{i=1}^{M_{k+1|k}}\frac{r_{k+1|k}^{\left(i\right)}p_{k+1|k}{}^{\left(i\right)}g_{k+1}\left(Z^{\left(i\right)}|x\right)p_{D,k+1}}{1-r_{k+1|k}^{\left(i\right)}\langle p_{k+1|k}{}^{\left(i\right)},p_{D,k+1}\rangle }}{\sum_{i=1}^{M_{k+1|k}}\frac{r_{k+1|k}^{\left(i\right)}\langle p_{k+1|k}{}^{\left(i\right)},g_{k+1}\left(Z^{\left(i\right)}|x\right)p_{D,k+1}\rangle }{1-r_{k+1|k}^{\left(i\right)}\langle p_{k+1|k}{}^{\left(i\right)},p_{D,k+1}\rangle }}$$

where $\langle a,b\rangle =\int_{\chi }a(x)b(x)dx$ denotes the inner product, and the sequential update processing is as shown

(35) $$r_{{}_{k+1}}^{}=\Psi_{k+1}{}^{\left(Ns\right)}\circ \cdots \circ \Psi_{k+1}{}^{\left(2\right)}\circ \Psi_{k+1}{}^{\left(1\right)}r_{k+1|k}^{\left(i\right)}$$

(36) $$p_{k+1}\left(x_{k+1}\right)=\Psi_{k+1}{}^{\left(Ns\right)}\circ \cdots \circ \Psi_{k+1}{}^{\left(2\right)}\circ \Psi_{k+1}{}^{\left(1\right)}p_{k+1|k}\left(x_{k+1}\right)$$

where ∘ denotes a composition.

### 3.2. State Extraction and Cardinality Biass

Extract multi-target states are the same as that of the GM-MB filter; for more details see [21]. The number of targets is estimated by:

(37) $${\tilde{N}}_{k}=\sum_{i=1}^{M_{k+1|k}}r_{L,k+1}^{\left(i\right)}+\sum_{z\in Z_{k}}r_{U,k+1}^{}(z)$$

For completeness, the key steps of the proposed filter are summarized as a block diagram of the processing algorithm in Table 1.

## 4. Experimental Results

### 4.1. Experimental Configuration

We developed the PBR system in Huazhong University of Science and Technology by tracking a close-in civilian airplane whose working frequency band is 6–30 MHz. The system is configured to work in multi-transmitter and receiver-only mode. The experiment was carried out in December 2014, in which three AM radio broadcast stations were selected as the noncooperative transmitters, namely, Tx1, Tx2, and Tx3, respectively. The specific parameters can be obtained from the International Telecommunication Union (ITU) Radiocommunication Sector [32,33] listed in Table 2, including carrier frequency (*f_c_*), transmitting power, distance with respect to the receiver, and so on.

**Figure 4:**
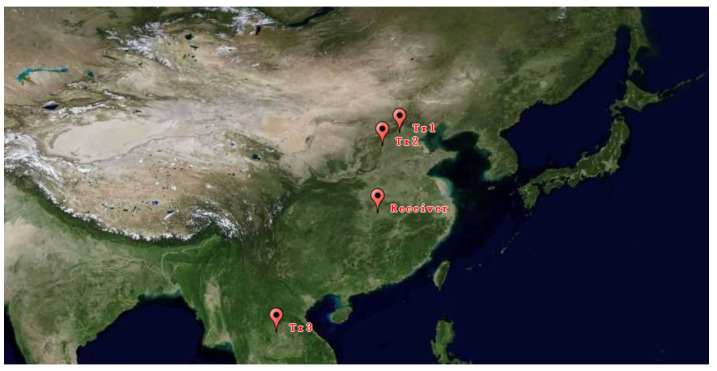
The geographical distribution of the illuminators and the receiver.

The ground distance between the AM radio broadcast station and the receiver antenna array is over 800 km. Thus, the transmitted signals are reflected by the ionosphere to reach targets over-the-horizon away. Figure 4 shows the geographical distribution of the illuminators and the receiver station.

**Figure 5:**
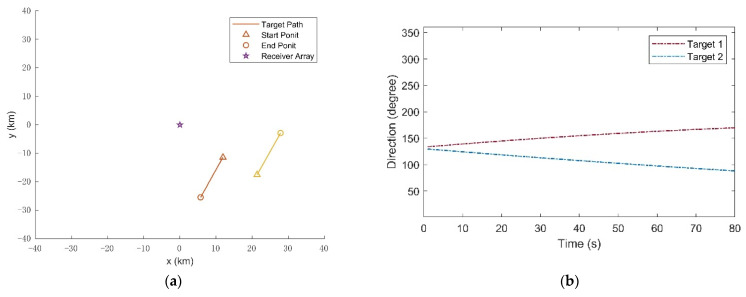
A part of civil aircraft parameters broadcasted by ADS-B in the experiment. (**a**) The real civil aircraft traces in x-y coordinate; (**b**) The direction of the civil aircrafts with respect to the receiver (based on the azimuth with respect to Eastern).

The noncooperative targets in the experiment are two civil aircrafts in the surveillance area with flight numbers CCAXXXX and CSNXXXX, respectively, namely, Target 1 and Target 2. The civil aircrafts parameters were broadcast by the automatic dependent surveillance-broadcast (ADS-B) system within a short interval of time. The data sets, including position, velocity, and so on, are the reference to verify the tracking method, which is recorded by a ground-based AirNav Radar Box. The two real trajectories of the civil aircrafts during the experiment are plotted in Figure 5a, which start represented by a triangle symbol and end represented by acircle symbol. As the recorded data show in Figure 5a, the two targets exist during the whole time in the surveillance. Target 1 flew at a constant altitude of 8.4 km with a nearly constant speed $v_{x}^{\left(1\right)}=106m/s$, $v_{y}^{\left(1\right)}=238m/s$, and Target 2 flew at a constant altitude of 5 km with a nearly constant speed $v_{x}^{\left(2\right)}=-87m/s$, $v_{y}^{\left(2\right)}=-196m/s$ in the surveillance area. The height of the transmitting station and the receiver are approximately ignored. Figure 5b plots the variation of direction of the targets during the experiment. The sampling interval was △ = 1 s, and the total experimental duration was 80 s.

### 4.2. Field Experimental Results

The targets are observed in the surveillance region with dimensions [−40,40]km ×[−40,40]km. The single-target transition model is a linear Gaussian process given by Equation (11), in which ∆ = 1 s is the sampling period, and $\sigma_{v}^{}=0.1{m/s}^{2}$ is the standard deviation of the process noise. The birth process is multiBernoulli with density $\pi_{\Gamma }={\left\{r_{\Gamma }^{\left(i\right)},p_{\Gamma }^{\left(i\right)}\right\}}_{i=1}^{2}$, where $r_{\Gamma }^{\left(i\right)}=0.01$,$p_{\Gamma }^{\left(i\right)}(x)=N(x;m_{\Gamma }^{\left(i\right)},P_{\Gamma })$,$m_{\Gamma }^{\left(1\right)}={\left[-20,000,0,20,000,0\right]}^{T}$, $m_{\Gamma }^{\left(2\right)}={\left[20,000,0,-20,000,0\right]}^{T}$, $P_{\Gamma }=diag{\left({\left[1000,10,1000,10\right]}^{T}\right)}^{2}$. The probability of target survival is $p_{S}{}_{,k}=0.95$. The probability of target detection is $p_{D,k}^{}=0.5$.

**Figure 6:**
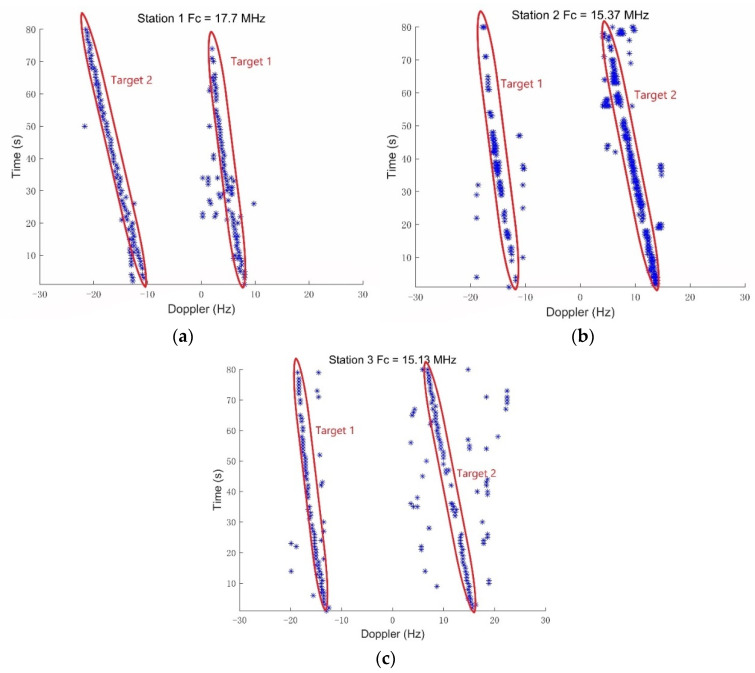
The results of target detection and Doppler shift measurements vs. time obtained from the surveillance areas using three AM broadcast stations. (**a**) Station 1 with a carrier frequency of 17.77 MHz; (**b**) Station 2 with a carrier frequency of 15.37 MHz; (**c**) Station 3 with a carrier frequency of 15.13 MHz.

**Figure 7:**
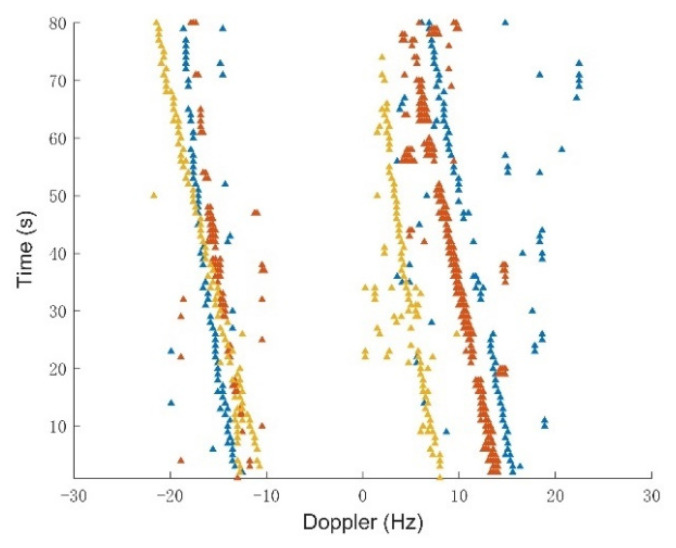
Doppler shift measurement set in which each color triangle stems from an AM broadcast transmitter.

After the aforementioned signal processing, we obtained the DOA estimation of each direct wave and the Doppler shift measurement data, including the false alarms and misdetections. Figure 6 shows the detected Doppler vs. time obtained from the surveillance areas using three AM broadcast stations with the carrier frequency of 17.7 MHz, 15.37 MHz, and 15.5 MHz, respectively. The Doppler measurement sets have clutter and the missing alarm. Then, the noisy three stations Doppler-shift measurement sets $Z_{1:80}^{\left(I\right)}$, are passed to the tracking filter, as plotted in Figure 7. The parameters of the tracking filter are set as follows: observation noise covariance $R_{k}=\sigma_{\varepsilon }^{2}I_{1}$, where $\sigma_{\varepsilon }^{}=1\mathrm{Hz}$ is the standard deviation of the measurement noise. Clutter parameter is Poisson with intensity $\kappa_{k}^{}(z)=\lambda_{c}Vu(z)$, where u(z) is a uniform probability density over the surveillance region, $V=1600{\;\mathrm{km}}^{2}$ is the “volume” of the surveillance region, and the clutter intensity is $\lambda_{c}^{\left(i\right)}=0.3$; *i* = 1,2,3 for z∈Z=[−30Hz,30Hz] are all assumed time invariant and independent of the target state.

**Figure 8:**
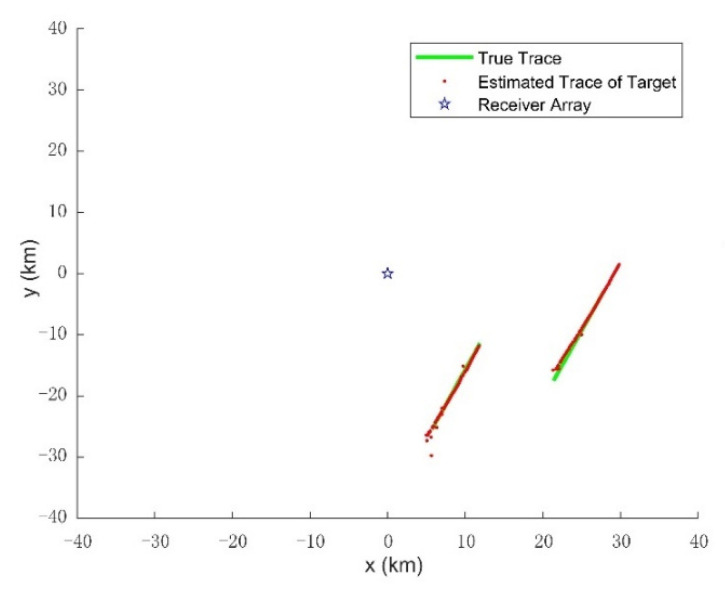
The estimated target path compared with the true path in x-y coordinate using 3 AM illuminators.

**Figure 9:**
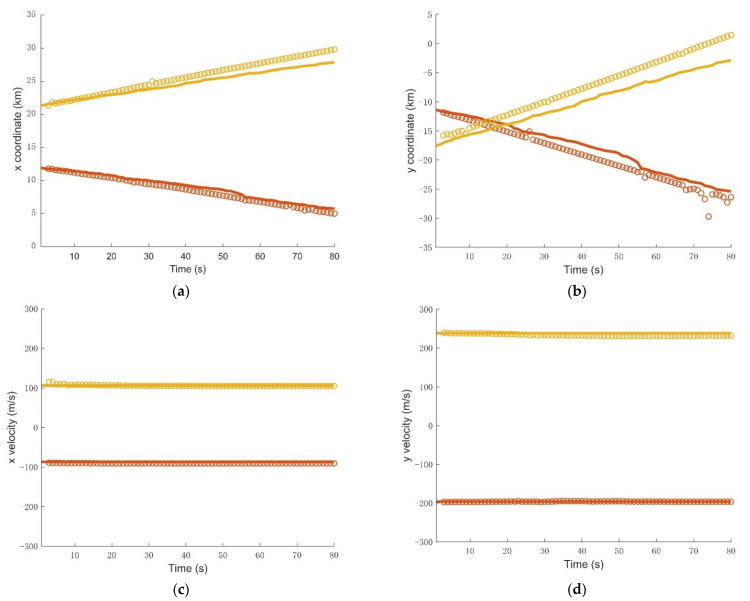
The estimated components of the state vector (dotted line) changes as functions of time compared with the true values (solid line). (**a**)The *p_x_* component; (**b**) The *p_y_* component; (**c**)The *v_x_* component; (**d**) The *v_y_* component.

**Figure 10:**
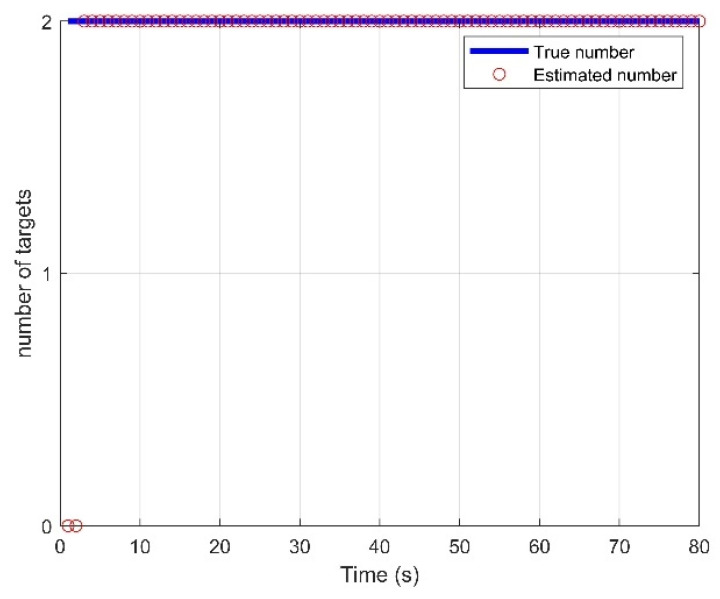
Estimated number of targets for the proposed method.

The initial density of the target state $p_{0}(x)$ is the Gaussian mixture of the form:$p_{0}(x)=\sum_{n=1}^{4}\omega_{0}N(x;m_{n},Q_{0})$,where $\omega_{0}=1/4$ is the weight factor of each initial state vector, $Q_{0}=diag([{\left(10^{3}m\right)}^{2},{\left(10m/s\right)}^{2},{\left(10^{3}m\right)}^{2},{\left(10m/s\right)}^{2}])$ is the initial covariance matrix, $m_{n,0}={\left[p_{xn,0},0m/s,\;p_{yn,0},0m/s\right]}^{T}$,n=1,2,…,4 are the initial mean state vectors, and $\left(p_{xn,0},p_{yn,0}\right)$ means four initial positions uniformly distributed in the surveillance area. In GM implementations, some of the parameters used in the filtering are: the target existence threshold $r_{k}=0.001$; at each time step, Gaussian components are pruned and merged for each hypothesized track with weight threshold *T* = 10^−10^ merging threshold U = 40, maximum components J_max_ = 200. In addition, hypothesized tracks are pruned with maximum *T_max_* = 5 and weight threshold *L* = 10^−5^. Tracking results of the IC-UT-GM-CBMeMBer filter are shown in Figure 8 and Figure 9, which show the estimated target traces and four estimated components of the state vector: *p_x_*, *p_y_*, *v_x_*, *v_y_* change vs time compared with the true trajectories, respectively. At ending time instants, short discontinuities occur in the tracks owing to the missing alarm of the Doppler measurement. Notice that the number of targets suffers from latency problem at the beginning of tracking in Figure 10, because the initial points are located arbitrarily.

**Figure 11:**
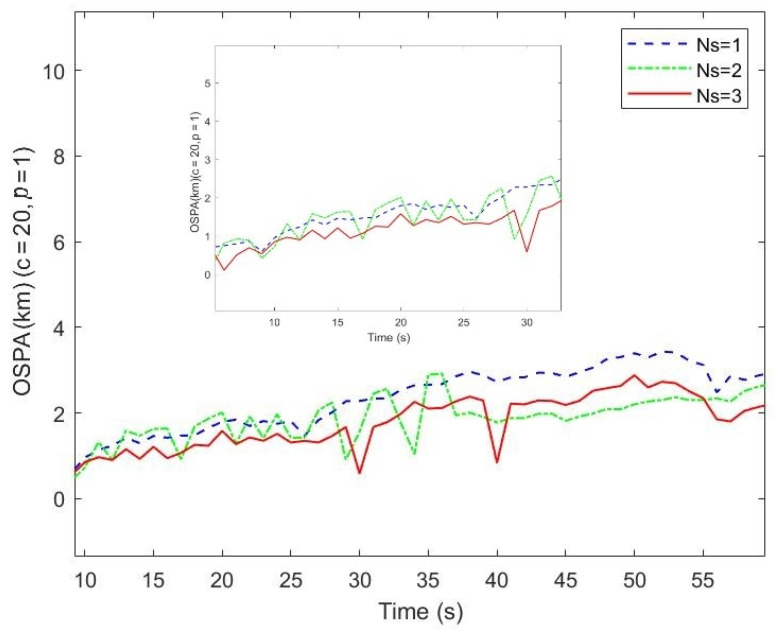
The OSPA distances vs. time on conditions of different station numbers.

Different numbers of illuminators are a problem in multi-target tracking performance when only Doppler measurements are used. To study this, the filter is implemented under the conditions of AM radio broadcast stations *Ns* = 2,1, corresponding to stations with serial numbers of *I* = {1, 2}, {1}, respectively. The optimal subpattern assignment (OSPA) is used to evaluate the tracking miss-distance. The OSPA distances (for *c* = 20 and *p* = 1) vs. time on conditions of various number of broadcast stations compared with the results on the condition of *Ns* = 3, *I* = {1, 2, 3} is shown in Figure 11. Particularly, the OSPA distances vs. the time between 6s and 32s is plotted. It can be seen that the estimated largest OSPA distances are approximately 2880 m, 2922 m, and 5712 m on the condition of *Ns* = 3, *Ns* = 2, and *Ns* = 1, respectively. The obvious error in the period time from *k* = 27 to 56 is due to the missing detections and clutter of the Doppler measurements and the number of illuminators. Therefore, we believe that the more numerous the AM radio broadcast stations that are exploited, the more accurate the tracking trajectories are.

### 4.3. Simulation Results

In this subsection, the performance of the proposed method is verified via simulation under similar scenarios to those aforementioned under the situation of cross trajectories, in consideration that it usually occurs in real data processing on a 2D Cartesian coordinate. Three AM broadcast stations were chosen, the same as Table 2. The two targets’ motion is assumed to be a nearly constant model adjusted for civil aircrafts, and the flight parameters are listed in Table 3. The false alarms are uniformly distributed in the field of view with range −30 Hz to 30 Hz, and the number of false alarms at each scan follows the Poisson distribution with a mean of 10. The parameters of the tracking filter are set the same as in Section 4.2.

**Figure 12:**
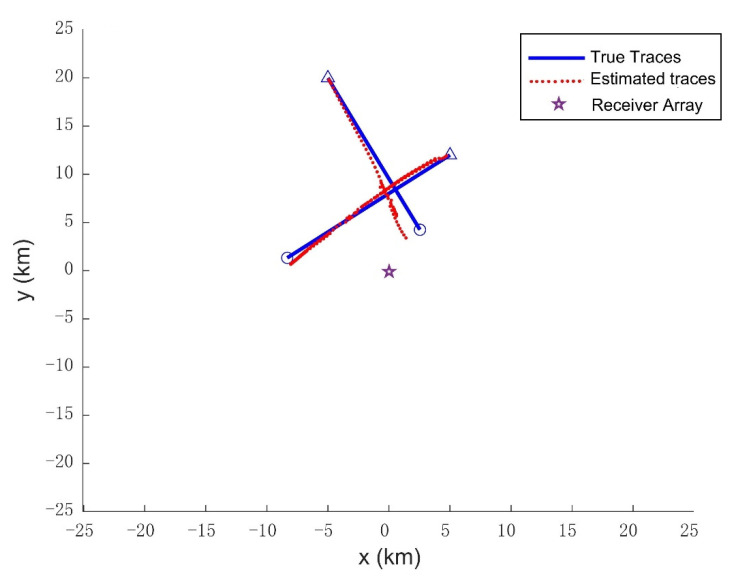
The estimated target path compared with the true path in x-y coordinate.

**Figure 13:**
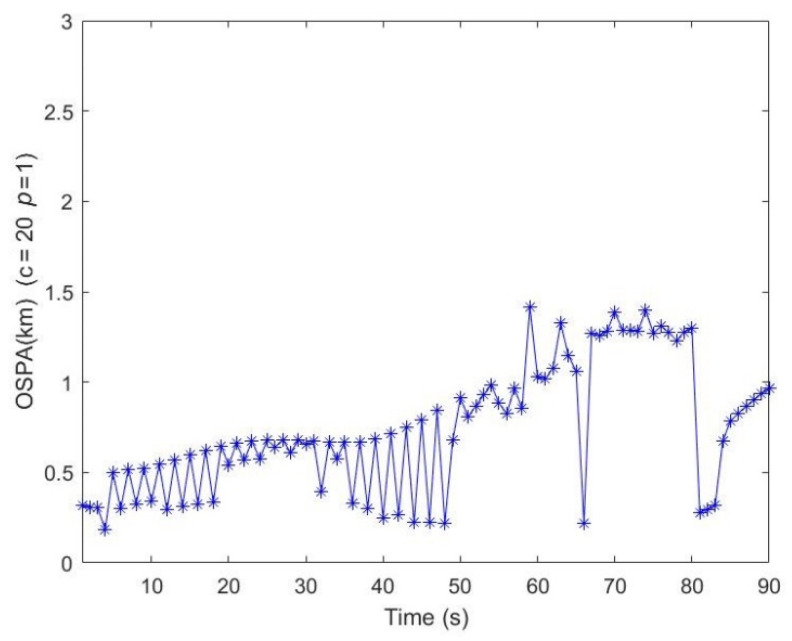
The OSPA distances vs. time under the situation of cross trajectories.

As shown in Figure 12, despite the intersection points, the two targets can follow their trajectories, respectively. In Figure 13, the OSPA metric (*p* = 1, *c* = 20) shows the track maintenance quality of the proposed method. However, the instantaneous peaks are observed from times *k* = 59 to *k* = 81 due to corresponding intersection point and track termination latency. The simulation results indicate that the proposed method can deal with relatively complex tracking problems.

## 5. Conclusions

In this paper, we propose a multi-target tracking filter in a self-developed PBR system by using spatially distributed multiple AM broadcast stations. Multiple non-cooperative illuminators with different carrier frequencies located over-the horizon and one receiver in the surveillance area are involved in the practical system. The direct wave and the illumination wave are reflected from the ionosphere received by a uniform circular array located over the horizon, while the echo wave (target-to-receiver) is via LOS propagation in the surveillance area. After some techniques in array signal processing, the Doppler measurement sets, including clutter and the missing alarm with corresponding time, can be collected. To overcome linear Gaussian models, we propose the tracking model and extend the GM-CBMeMBer filter to a practical nonlinear measurement model by using unscented transform (UT) techniques by iterating the filter update stage for each illuminator measurement set in this practical scenario. Three AM broadcast stations were selected as the non-cooperative illuminators. Two non-cooperative civil aircrafts were chosen as tracking targets, whose flight parameters were recorded by a ground-based AirNav Radar Box set. Considering the clutter and missing alarm in the measurement sets, the OSPA distances are acceptable. Moreover, the performance of simulation has verified the feasibility of the proposed tracking method. In future work, the unknown clutter rate and detection probability under unknown background in this practical scenario will be taken into consideration. Maneuvering target tracking is also worthy of study.

## Figures and Tables

**Table 1:** Pseudocode of the proposed filter.

$\mathrm{Input}:\;\mathrm{Initial}\;\mathrm{Gaussian}\;\mathrm{Mixtures}\;{\left\{w_{0},m_{0},P_{0}\right\}}_{i=1}^{J_{0}}$ $\;\mathrm{and}\;\mathrm{Doppler}\;\mathrm{Measurement}\;\mathrm{Set}\;Z_{1:k}^{\left(I\right)}$
for *k* = 1:Time duration predict surviving Gaussian components for *i* = 1:*M_k_*
$r_{P,k+1|k}^{\left(i\right)}=r_{k}^{\left(i\right)}p_{S,k+1}$ for *j* = 1:*J_k_* $\mathrm{compute}\;m_{P,k+1|k}^{\left(i,j\right)}=F_{k}m_{k}^{\left(i,j\right)}$$P_{P,k+1|k}^{\left(i,j\right)}=Q_{k}+F_{k}P_{k}^{\left(i,j\right)}F_{k}^{T}$ end end
construction of birth target Gaussian components using Equation (13) end update the legacy tracks for *i* = 1:*M_k_*_+1|*k*_ $r_{L,k+1}^{\left(i\right)}=r_{k+1|k}^{\left(i\right)}\frac{1-p_{D,k+1}}{1-r_{k+1|k}^{\left(i\right)}p_{D,k+1}}$$m_{L,k+1}^{\left(i\right)}=m_{L,k+1|k}^{\left(i\right)}$$P_{L,k+1}^{\left(i\right)}=P_{L,k+1|k}^{\left(i\right)}$ end
update the measurement-corrected tracks for freq = 1:*Ns* for *i* = 1:$M_{k}^{freq}$ for *j* = 1: M_k+1|k_ $\mu_{k+1}^{\left(j\right)}=\left[\begin{array}{l} m_{k+1|k}^{\left(j\right)}\\ 0\\ 0 \end{array}\right]$$C_{k+1}^{\left(j\right)}=\left[\begin{array}{l} P_{k+1|k}^{\left(j\right)}\\ Q_{k+1}\\ R_{k+1} \end{array}\right]$ each component constructs a set of sigma points and weights using Equation (22) to generate: ${\left\{\chi_{k}^{\left(\ell \right)},u^{\left(\ell \right)}\right\}}_{\ell =0}^{L}$ $z_{k+1|k}^{\left(\ell \right)}=h_{k}(\chi_{k+1}^{\left(\ell \right)}),\;\ell =0,\ldots ,L$$h\left(\chi_{k}^{\left(\ell \right)}\right)=\frac{f^{\left(freq\right)}}{c}\left[-\vec{v_{k}^{\left(\ell \right)}}{\vec{p}}_{k}{}^{\left(freq\right)}+\vec{v_{k}^{\left(\ell \right)}}\cdot {\vec{vt}}_{k}{}^{\left(freq\right)}\right]$ end $\mathrm{compute}\;\{(r_{U,k+1},p_{U,k+1})\}$ using Equations (18) and (19) end prune tracks end state extraction and cardinality bias using Equation (37) end

**Table 2:** Parameters of the three AM radio broadcast stations used as transmitters.

Station Serial Number	f_c_ (*k*Hz)	Station Name	Power (kW)	Latitude (deg)	Longitude (deg)	Distance (km)
Tx1	17770	UDO	250	17.25	102.48	1866
Tx2	15370	SZG	100	38.04	114.28	842
Tx3	15130	BEI	150	39.55	116.25	1027

**Table 3:** The flight parameters in simulation.

Item	Initial Position (km)	Initial Velocity (m/s)	Time of Birth (s)	Time of Death (s)
Target 1	(−5,20)	(95, −200)	1	80
Target 2	(5,12)	(−150, −120)	1	90

## Data Availability

Not applicable.
